# The nosological significance of Folie à Deux: a review of the literature

**DOI:** 10.1186/1744-859X-5-11

**Published:** 2006-08-08

**Authors:** Danilo Arnone, Anish Patel, Giles Ming-Yee Tan

**Affiliations:** 1 Department of Psychiatry, Warneford Hospital, Oxford, UK; 2 Division of Mental Health, St George's University of London, London, UK; 3 Department of Psychiatry, West Sussex Health and Social Care NHS Trust, Worthing, UK; 4 Department of Psychiatry, University of Southampton, Southampton, UK

## Abstract

**Background:**

Folie à Deux is a rare syndrome that has attracted much clinical attention. There is increasing doubt over the essence of the condition and the validity of the original description, such that it remains an elusive entity difficult to define.

**Method:**

We conducted a systematic review of the literature of all cases reporting the phenomenon of Folie à Deux, from the years 1993–2005.

**Results:**

64 cases were identified of which 42 met the inclusion criteria. The diagnoses in the primary and secondary were more heterogeneous than current diagnostic criteria suggest. There exists a high degree of similarity between the primary and secondary in terms of susceptibility to psychiatric illness, family and past psychiatric history, than previously thought.

**Conclusion:**

Folie à Deux can occur in many situations outside the confines of the current classification systems and is not as rare as believed, and should alert the clinician to unrecognized psychiatric problems in the secondary.

## Background

Lasègue and Falret [1] first described the phenomena of the transference of delusional ideas from a 'primary' affected individual to one or more 'secondaries', in close association. They coined the term 'Folie à Deux', a relatively rare syndrome that has long since attracted much clinical attention. Although 'Folie à Deux' is probably the most widely used term for this type of disorder, many other terms are used synonymously such as 'double insanity' and 'psychosis of association', leading to considerable confusion. The early criteria for 'Folie à Deux' outlined by Lasègue and Farlet assumed some aetiological factors that significantly shaped subsequent psychiatric thought, with little supporting evidence or critical examination (Table 1). Since the introduction of validated diagnostic criteria, very little has changed in the description of the phenomena. Standardised criteria adopt two main terms 'Induced delusional disorder' (ICD-10) [2], and 'Shared psychotic disorder' (DSM-IV) [3]. The principal limitation of such definitions is that they describe phenomena initially formulated in a milieu of societal values and psychodynamic views of a different era. This was mainly because a priori assumptions, impregnating the description of this phenomenon and incorporated into operational definitions, are difficult to test. To complicate matters even further, there have been attempts to organize the disorder into subtypes according to the psychopathology encountered [4]. For example terms like 'Folie Imposee', 'Folie Simultanee', 'Folie Communiqué' and 'Folie Indiute' designate subtypes of the phenomena of 'Folie à Deux' [5]. The lack of clarity is undoubtedly supported by objective limitations in the aetiological understanding of the syndrome, its rarity, as well as the limited knowledge of its natural history and prognosis. In the last century, a plethora of reports have been published. This has contributed to an increase in our knowledge of the neuropsychological mechanisms underlying the phenomena, beyond pure phenomenological descriptions. This has inevitably led to questioning not just the actual essence of the condition but also the validity of the original description in a way that it now resembles an elusive entity. A review [6], that adopted operational criteria in the identification of caseness, found discrepancies in the original description of this phenomenon. This work included all the published literature from 1942 to 1993 and revealed a substantial shift in the psychosocial aspects of the presentation of 'Folie à Deux'. It also described a high level of psychiatric morbidity in the secondary accompanied by an extensive family history for psychiatric illness. In this context exposure to the primary could act as a psychosocial trigger for a 'transient psychotic phenomenon' in a subject who would have developed a psychotic episode in any case. In previously published work, we showed that separation of the dyad doesn't always result in disappearance of psychopathology [7]. This is highly suggestive of a biological contribution to the condition. The possibility of psychiatric morbidity in the associate is absent in the description of the syndrome and doesn't appear in current diagnostic criteria [7]. Regarding the primary, the authors found a broader range of psychiatric conditions than what was originally described [6]. We propose a broader nosological entity than the one described by Lasègue. This includes a wider range of psychiatric conditions in the primary, the possibility of psychiatric morbidity in the secondary, susceptibility in the secondary not necessarily limited to gender differences, and the acknowledgement of socio-cultural changes in the presentation of this condition. We tested this hypothesis by extending the above-mentioned review from 1993 to 2005.

**Table 1:** Lasègue-Falret syndrome

A syndrome prevalent among women living more or less confined, marked by:
Coincidental appearance of psychotic symptoms in members of a family while living together
Appearance of psychotic symptoms in two closely associated persons
Transmission of psychotic symptoms from a sick person to one person or several healthy individuals who elaborate on the induced delusions

## Method

This article was intended to update the review conducted by Silveira and Seeman [6]. The scope of Silveira and Seeman's work was to identify cases of shared psychotic disorder, to extrapolate vital information from the literature and reframe the condition according to a more modern bio-psychosocial approach. The review also analysed in detail psychopathology at presentation. Articles were identified if presented original data, and addressed cases of induced delusional disorder, shared delusional disorder and 'Folie à Deux'. A comprehensive search from a range of databases including BNI, CancerLit, Cochrane Library, EMBASE, Medline, Psychoinfo and Pub Med was conducted from the end of 1993 to 2005. The search was also complemented by manual search of bibliographic cross-referencing. There was no restriction on the identification of studies in terms of publication status, language or design type. Key words used to identify the studies were: INDUCED DELUSIONAL DISORDER, SHARED DELUSIONAL DISORDER, and FOLIE A DEUX. We adopted criteria for inclusion similar to Silveira & Seeman [6]: clear discrimination between primary and secondary patients, demographic variables (age and gender), diagnosis according to diagnostic criteria including co-morbidity, past psychiatric history, family history, social isolation and other vulnerability factors, the nature of the relationship between primary and secondary and its duration, psychopathology, length of exposure to the primaries' psychosis. Management options were also included. The review excluded cases where more than one secondary was involved. We adopted a standardized assessment sheet (available upon request) to identify suitable articles and resolved disagreement by consensus.

## Results

**Figure 1:**
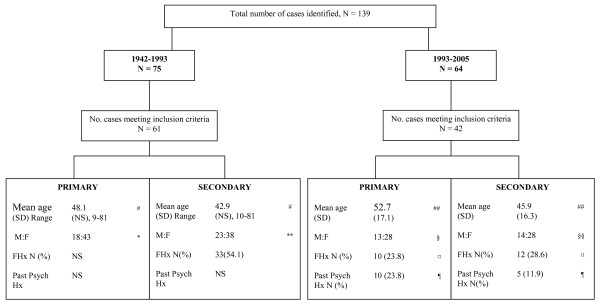
**Demographic Characteristics, Family and Psychiatric History**. Difference in age between primary and secondary: (#) not statistically significant difference; (##): χ^2 ^= 7.51, df = 26, p > 0.05. Difference in gender in the primaries: (*) χ^2 ^alpha = 0.005 df = 1 and (§) χ^2 ^= 4.67, df = 1, p > 0.05. Difference in gender in the secondaries: (**) χ^2 ^alpha = 0.05 df = 1 and (§§) χ^2 ^= 4.67, df = 1, p > 0.05. Difference in family history between primaries and secondaries: (¤)χ^2 ^= 0.84, df = 1, p = 0.359. Difference in past psychiatric history between primaries and secondaries: (¶) χ^2 ^= 1.67, df = 1, p = 0.196. NS = Not stated.

A total of 64 published cases were identified of which 42 [8-41] met the criteria for inclusion (Figure 1). Findings (years 1993–2005) were compared with those from Silveira and Seeman's review [6] (years 1942–1993). The information is divided into the following categories accompanied with the relevant figures and tables for ease of explanation.

### Demographic characteristics, family and psychiatric history (Figure 1)

*1) Age*: In the years 1993–2005 age was reported in 37 (88%) of primary and 33 (79%) of secondary cases. Mean age was 52.7 for primaries (SD = 17.07) and 45.9 (SD = 16.3) for secondaries. This difference was not statistically significant (p > 0.05). In the years 1942–1993 the mean age of the primaries was 48.1 years and for the secondaries 42.9 years. This difference was not statistically significant. *2) Gender*: In the years 1993–2005 gender was reported in 41 (98%) of primaries (13 men and 28 women) and 42 (100%) of secondaries (14 men and 28 women). The difference between men and women in the primaries and secondaries was not statistically significant (p > 0.05). In the years 1942–1993, gender was identified in all primaries and secondaries (N = 61, 100%). Primaries consisted of 18 men and 43 women, and secondaries of 23 men and 38 women. Whilst within the group of secondaries gender differences were not statistically significant (p = 0.05), authors recorded an excess of women in the primaries which reached statistical significance (p = 0.005). *3) Family History*: In the years 1993–2005, family psychiatric history was reported in only 10 (23.8%) of primaries and 12 (28.6%) of secondaries. The difference between the two groups did not reach statistical significance (p = 0.359). Only the family history of secondaries was considered in the years 1942–1993 and was recorded in 33 (54.1%) of the subjects. *4) Psychiatric History*: Evidence of psychiatric history preceding the appearance of 'Folie à Deux' was reported in 10 primaries (23.8%) and 5 secondaries (11.9%). The difference between primary and secondaries was not statistically significant (p = 0.196). This information was not elicited in the years 1942–1993.

### The nature of the relationship (Table 2)

**Table 2:** The nature of the relationship

**Nature of the relationship**	**1942–1993 **N = 61(%)	**1993–2005 **N = 42 (%)
Husband:Wife	10 (16.4)	12 (28.6)
Wife:Husband	8 (13.1)	10 (23.8)
Parent:Child	19 (31.1)	6 (7.1) (Mother:Daughter = 5) (Mother:Son = 1)
Child:Parent	0	2 (4.2)
Siblings	18 (29.5), 3 Twins	11 (26.2), 5 Twins (all F) (Sisters = 10, Brothers = 1)
Friends	6 (9.9)	1 (2.4)

In the years 1993–2005 the type of relationship between primaries and secondaries was always well described (N = 42, 100%). The majority of the relationships were within the nuclear family (97.6%). The remainder occurred within the context of friendships (2.4%) between genetically unrelated individuals. The largest proportion was constituted by married or common-law couples (52.4%) with a similar distribution between husband and wife. The second largest group (23.8%) was between sisters (50% twins). Results emerging from 1942–1993 review also showed an increased susceptibility of the condition within the family. However, the dyad parent-child contributed more significantly (N = 19, 31.1%). These authors specified that offspring were secondaries in 73.7% of cases highlighting a possible increased susceptibility in child inductees. Siblings (sisters) and common law couples then followed (N = 18, 29.5%).

### Risk factors, duration of the association, and exposure in the dyad (Table 3)

**Table 3:** Risk factors, duration of the association, and exposure in the dyad

	**1942–1993 **N (%)	**1993–2005 **N (%)
**Dyad Risk Factors**	61 (100)	42 (100)
**N (%)**	Social Isolation: 53 (84)	Social Isolation 27 (64.3)
	Others in the secondaries: NS	Others in the secondaries:
		Secondary passive 5 (5.9)
		Cognitive impairment 3 (3.5)
		Language difficulties 1 (1.2)
		Life events 1 (1.2)
**Duration of the association (Months)**	47 (77.0)	32 (52.4)
**Mean (SD), Range**	NS (NS) 3–948	30.7 (19.07), 0.5–81.00
**Duration of the exposure (Months)**	NS	35 (57.4)
**N (%)**	NS	72.98 (86.46), 0.00–336.00

NS: Not stated

Social isolation has often been described as a major risk factor for the development of 'Folie à Deux'. Social isolation was reported in 64.3% (N = 27) of the cases identified in the years 1993–2005 and 84% (N = 53) in the years 1942–1993. A number of other factors were also reported in the secondaries in more recent years: passive personality, cognitive impairment, language difficulties, and life events.

The duration of the association between primary and secondary was found to be in the range of several months in both 1993–2005 (0.5–81.00 months) and 1942–1993 (3–948 months). Only in the years 1993–2005 the length of the exposure was recorded with a mean of 72.98 months (SD = 86.46) suggestive of a long exchange of social interaction between primary and secondaries.

### Diagnosis, co-morbidity and psychopathology (Table 4)

**Table 4:** Diagnosis, co-morbidity and psychopathology

	**Diagnosis **N (%)	**Abnormal thoughts **Type, N, (%)	**Abnormal perceptions **Type, N, (%)
**1942–1993**			
**Total N**	54(88.5)	54 (88.5)	58 (95.0)
**Primary**	Schizophrenia 24 (44.4)	Delusions:	Hallucinations:
	Mood disorders 7 (13.0)	Persecutory 46 (75.4)	Type clearly described 30 (52.6)
	Delusional disorders 6 (11.1)	Grandiose 8 (13.1)	Not sufficient data 18 (47.4)
**Secondary**	Pure Shared delusional disorder 54 (88.5)	Delusions:	Hallucinations:
	Co-morbidity 48 (89.0):	No difference in type or quality reported.	Same type but less intense quality 17 (29.3)
	Dementia		Only in the secondary 2 (3%)
	Depression		No sufficient data 39 (67.0)
	Mental retardation		
**1993–2005**			
**Total N**	45 (100)	53 (87)	25 (59.5%)
**Primary**	Delusional disorder 15 (33.3)	Delusions:	Hallucinations:
	Schizophrenia 13 (28.9) Depression 3 (6.7) Bipolar affective disorder 3 (6.7) Induced delusional disorder 3 (6.7) Mixed affective disorder 1 (2.2) Psychosis (NOS) 1 (2.2) Mixed affective disorder 1(2.2) Mania 1 (2.2) OCD 1 (2.2) Cognitive impairment 1 (2.2)	Persecution 30 (35.3) Grandiose 13 (15.3) Erotomanic 4 (4.7) Somatic 3 (3.5) Infestation 1 (1.2) Capgras' 1 (1.2) Others: Obsessions and compulsions 1 (1.2)	Auditory 11 (45.8) Somatic 5 (20.8) Visual 4 (16.6) Tactile 3 (12.6) Olfactory 1 (4.2)
**Secondary**	Pure Induced delusional disorder 30 (71.4) Co-morbidity 12 (28.6): Schizophrenia 6 (14.3) Depression 3 (7.1) Cognitive impairment 1 (2.4) Bipolar affective disorder 2 (4.8)	Delusions: No difference in type between primaries and secondaries. The intensity of the experiences was also identical in 98% of cases. In the remaining 2% secondary had less intense abnormal thoughts.	Hallucinations: Same type but less intense quality 13 (52.0%) As primary 11 (44.0) Only in secondary 1 (4.0)*

(*) Haptic hallucinations.

*1) Diagnosis in the Primary*: In 1993–2005, the diagnosis was recorded in all the cases retrieved (N = 42, 100%). The commonest diagnosis in the primary was delusional disorder followed by schizophrenia and affective disorders. The diagnosis in the years 1942–1993 was recorded in 54 cases (88.5%), but the order of frequency was different with schizophrenia first followed by mood disorders and delusional disorders. *2) Diagnosis and Co-morbidity in the Secondary*: In the secondary, 'Folie à Deux' was the primary diagnosis in 71.4 % of cases (N = 30) in 1993–2005 and 88.5% (N = 54) in 1942–1993. However other diagnosis were also highly represented. In 1993–2005 schizophrenia was recorded 6 times (14.3%), followed by depression (N = 3, 7.1%), cognitive impairment (N = 1, 2.4%) and bipolar affective disorder (N = 2, 4.8%). Similarly in the years 1942–1993 a co-morbid diagnosis in the secondary was recorded in 89.0% of cases (N = 48) and included depression, dementia and mental retardation. *3) Psychopathology*: In the primary, delusions were commonly recorded in both 1942–1993 (N = 54, 88.5%) and 1993–2005 (N = 53, 87%). Persecutory and grandiose delusions were most commonly encountered. Notably, in the majority of cases (98–100%), delusions were identical in the dyads. In 1993–2005 cases only 2% of secondaries experienced less intense phenomena and one case of obsessive thoughts was also reported. In terms of hallucinations, much more variability was described and, in general, secondaries had a less intense experience in a large number of cases both in 1993–2005 (N = 13, 52.0%) and 1942–1993 (N = 17, 29.3%). Frequency of hallucinations was higher in 1942–1993 (N = 58, 95.0%) compared to 1993–2005 (N = 25, 59.5%) but the quality was not always sufficient to allow a detailed description. When described, auditory hallucinations were the commonest, followed by somatic and visual. In 1993–2005 one of the secondaries developed hallucinations not shared by the primary.

### Management (Tables 5 and 6)

**Table 5:** Treatment options

	**Primary **N (%)	**Secondary **N (%)
**Separation only**	5 (11.9)	8 (19.0%)
**Separation and medication**	14 (33.3)	13 (30.9)
**Medication alone**	30 (71.4)	26 (61.9)
	Antipsychotics 15 (35.7)	Antipsychotics 9 (21.4)
	Antidepressants 1 (2.4)	Antidepressants 2 (4.8)
	Non specified 14 (33.3)	Non specified 15 (35.7)
**Medication in combination**	5 (11.9)	2 (4.8)
	Mood stabilisers/antipsychotics 4 (9.5)	Mood stabilisers/antipsychotics 2 (4.8)
	Antidepressants/antipsychotics 1 (2.4)	
**Psychotherapy**	2 (4.8)	6 (14.3)
**ECT**	1 (2.4)	0 (0)
**Others**	0 (0)	1 (2.4)*

(*) Cardio conversion

**Table 6:** Treatment settings

	**Primary **N (%)	**Secondary **N (%)
**Inpatient**	26 (61.9)	22 (52.4)
**Outpatient**	4 (9.5)	7 (16.6)
**No Follow up**	4 (9.5)	4 (9.5)
**Death**	1 (2.4)	1 (2.4)

Information on management was only available in the years 1993–2005 and extensively involved separation of the dyad in most cases. This intervention was commonly used in combination with medication in both primary (N = 14, 33.3%) and secondary (N = 13, 30.9%). Use of medication was frequently used alone or in combination. In particular, antipsychotics and antidepressants were widely used. In combination, mood stabilisers and antipsychotics were the most successful in both primary and secondary, followed by antipsychotics and antidepressants in one primary only. Treatment settings when reported involved inpatient management in a large number of cases for both primary (N = 26, 61.9%) and secondary (N = 22, 52.4%), followed by outpatient interventions, absence of follow up and premature death.

## Discussion

Current diagnostic criteria highlight the importance of the absence of psychiatric illness in the secondary other than psychopathology induced by close proximity with the primary. Data from case studies shows that this presumed hypothesis is not always applicable. Secondaries show a high comorbidity rate which ranges between 28.6–89.0%. This finding questions the validity of the diagnosis and supports the possibility that the close proximity of primary and secondary only constitute a temporal trigger for a psychiatric condition in already susceptible individuals, who would have developed a psychiatric disorder anyway. This is also supported by the high rate of psychiatric morbidity in the secondary also in terms of family history and past psychiatric history. Family psychiatric history was in fact present in the secondaries in 28.6–54.1% of cases. In the years 1993–2005, there was no statistically significant difference between the primary and the secondary in terms of the family history (p = 0.359). This suggests a similarity in genetic loading for psychiatric disorders between the primary and the secondary. Similarly, there was evidence of a positive psychiatric history preceding the appearance of the disorder in both the primaries and the secondaries although the difference was not statistically significant (p = 0.196). The information supports a high degree of similarity between primaries and secondaries in terms of susceptibility to psychiatric illness. The common belief highlighted by diagnostic criteria that 'separation' as the means of treatment of the secondary is the only and sufficient intervention required is also not supported in this review. Secondaries were extensively treated with medication to similar levels of the primaries, often in conjunction with separation and other interventions (e.g. psychotherapy), revealing that separation is not always the treatment of choice for the secondaries. Among predisposing factors linked to the development of the disorder, social isolation was reported very frequently between 64.3% and 84%. However, a number of other factors were also reported like passive personality, cognitive impairment, language difficulties, and life events. This is partly in keeping with current diagnostic criteria but also expands on the possible existence of other factors which constitute a disadvantage for the secondary which are not necessarily related to the primary. Some of these factors can be considered predisposing factors for mental illness in their own right. The diagnosis in the primaries was found to be more heterogeneous than current diagnostic criteria suggest. Although schizophrenia is indeed very common, delusional disorder, and affective disorders were also commonly seen, implying that a wider inclusion criteria could be adopted. In terms of the content of delusions, persecutory and grandiose were very common but there was a plethora of other different types. Furthermore, hallucinations, which are currently not included in the diagnostic criteria occur frequently and therefore could be included in future versions of a diagnostic classification system.

### Limitations

Limitations in this study include the variability of diagnostic criteria (e.g. ICD 10 and DSM IV) used in the identification of caseness which although standardised are not entirely identical. If we had adopted non-standardised criteria for the identification of the disorder, it would have led to over-inclusion of numerous reports, thus limiting the validity of the review. Finally, although the inclusion-exclusion criteria adopted by this article were tailored on Silveira and Seeman's review [6], there may be some further methodological differences which allowed only comparison but not combination of data. For instance, we adopted a more inclusive searching strategy to minimize publication bias and we dealt with disagreement by consensus. With all the limitations, this article offers the largest and most up to date review of cases of 'Folie à Deux' ever published and supports a possible variability in the condition and emphasises the necessity of further work in the future.

## Conclusion

We set out to propose a broader nosological entity than that originally described by Lasègue in 1877 [1] on the phenomenon of 'Folie à Deux'. We carried out a review of all cases reporting the phenomenon of 'Folie à Deux' from the years 1993–2005, which followed on from an earlier review by Silveira and Seeman [6]. Our findings on the whole were in keeping with those of Silveira and Seeman's, and suggest that the phenomenon of 'Folie à Deux' first described in 1877 and the criteria necessary to make its diagnosis within current classification systems to be insufficient. The data shows that the primary need not only have schizophrenia to induce shared psychotic symptoms in the secondary, but that a variety of other mental illnesses could also be responsible. The shared psychotic symptoms themselves need not only be delusions but also be hallucinations. Secondaries, traditionally described to have a submissive role in the dyad but otherwise mentally sound, could actually be extremely vulnerable to developing or having a significant mental illness themselves. The treatment that is often advocated ie. separation, has also been shown to be inadequate or insufficient in a large number of cases. We hope these findings make clinicians aware that the phenomenon that is essentially the transfer of psychotic symptoms from one (primary) individual to another (secondary) can occur in many situations outside of the confines of the current diagnostic classification systems and therefore is perhaps not as rare as is believed; and that the occurrence of shared psychotic symptoms should alert the clinician to further investigate the presence or monitor the development of any mental illness in the secondary as this may go unrecognised.

## Competing interests

DA has received bursaries from Janssen-Cilag Ltd.

### Clinical implications

• Folie à Deux can occur in many situations outside the confines of current diagnostic classification systems, and is perhaps not as rare as is believed.

• The occurrence of the phenomenon of Folie à Deux should alert the clinician to investigate the presence or monitor the development of psychiatric illness in the secondary.

• Separation is often the treatment option most advocated, but it may be inadequate or insufficient.

### Limitations

• There was variability of the diagnostic criteria (i.e. ICD-10 and DSM-IV) used in the identification of cases which although standardised are not entirely identical.

• Due to methodological differences in the collection of data we were unable to combine our results with an earlier review and could only draw comparisons to it.

• The review was based on data that was not critically appraised as it was collected
